# The link between plant‐based diet indices with biochemical markers of bone turn over, inflammation, and insulin in Iranian older adults

**DOI:** 10.1002/fsn3.2258

**Published:** 2021-03-29

**Authors:** Hossein Shahinfar, Mohammad Reza Amini, Nastaran Payandeh, Sina Naghshi, Fatemeh Sheikhhossein, Kurosh Djafarian, Sakineh Shab‐Bidar

**Affiliations:** ^1^ Department of Nutrition School of Public Health Iran University of Medical Sciences Tehran Iran; ^2^ Department of Clinical Nutrition Faculty of Nutrition Sciences and Food Technology Shahid Beheshti University of Medical Sciences Tehran Iran; ^3^ Department of Community Nutrition School of Nutritional Sciences and Dietetics Tehran University of Medical Sciences (TUMS) Tehran Iran; ^4^ Department of Clinical Nutrition School of Nutritional Sciences and Dietetics Tehran University of Medical Sciences (TUMS) Tehran Iran

**Keywords:** bone metabolism marker, inflammation, insulin resistance, plant‐based diet index

## Abstract

**Background:**

The association of plant‐based diets and biomarkers of bone, insulin, and inflammation is still unclear.

**Objectives:**

We investigated the associations between biomarkers of bone, insulin, and inflammation and three plant‐based diet indices: an overall plant‐based diet index (PDI); a healthy plant‐based diet index (hPDI); and an unhealthy plant‐based diet index (uPDI).

**Methods:**

We included 178 elderly subjects who referred to health centers in Tehran. Blood and urine samples were collected to measure osteocalcin. The Human C‐telopeptide of type Ⅰ collagen (u‐CTX‐I), highly sensitive C‐reactive protein (hs‐CRP), parathyroid hormone (PTH), 25(OH) D, and insulin resistance and sensitivity. We created an overall PDI, hPDI, and uPDI from semi‐quantitative food frequency questionnaire (FFQ) data.

**Results:**

Dietary groups of Vegetables (*r *= .15, *p* = .03), nuts (*r *= .16, *p* = .03), dairy (*r *= .25, *p* = .001), eggs (*r *= .27, *p* < .001), red meat, and animal products (*r *= .25, *p* = .001) were directly correlated with osteocalcin. Refined grains were also had a positive association with serum insulin concentration (*r *= .14, *p* = .04). PTH levels are inversely associated with PDI score (β = −0.18, *p* = .01). Also, serum insulin concentration was negatively associated with PDI score (β = −0.10, *p* = .04). Urine CTX‐1 levels were significantly associated with hPDI score (β = −0.06, *p* = .04). u‐CTX‐1 levels are inversely associated with uPDI score. This significance did not change with the adjustment of the confounders (β = −0.28, *p* < .001).

**Conclusions:**

More adherence to PDI and hPDI and less in uPDI may have a beneficial effect on biomarkers of bone, inflammation, and insulin thus preserving chronic diseases.

## Graphical abstract

Plant‐based dietary pattern may have influence on several chronic diseases; however, there is no study that investigates the beneficial effect of plant‐based dietary pattern on biochemical markers of bone turn over, inflammation, and insulin on Iranian older adults. We found that adherence to plant‐based dietary pattern and healthful plant‐based dietary pattern have lucrative consequences on bone turn over, inflammation, and insulin biomarkers.

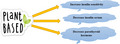

## 1 INTRODUCTION

Osteoporosis is a progressive skeletal disease that is characterized by low bone mass and loss of bone mineral density (BMD), which may lead to a greater risk of bone fractures, mainly in the wrist, spine, and hip. Osteoporosis and other bone diseases affect more than 10 million people and causing nearly 1.5 million fractures related to osteoporosis per year in the United States (Kitchin & Morgan, 2007; US, 2004). Women are at a higher risk of osteoporosis than men because of their smaller bone structure, hormonal changes, and pattern of bone loss (Johnell & Hertzman, 2006; Martin et al., 2004; Vondracek & Hansen, 2004). Along with early diagnosis and treatment, prevention and lifestyle changes that include physical activity and nutrition can help decrease a large portion of the harmful effects of bone disease (New et al., 1997). The bone formation process needs a sufficient and constant amount of mineral and nutrients, including calcium, protein, magnesium, phosphorus, vitamin D, and potassium (Tucker et al., 1999).

Plant‐based diets, such as vegan and vegetarian diets, are sort of eating patterns that emphasize legumes, unrefined grains, vegetables, fruits, nuts, and seeds and reduction or elimination of most or all animal products and are especially potent in inhibiting type 2 diabetes (T2D), which is a predominant consequence of insulin resistance (Dinu et al., 2020; Qian et al., 2019). Also, there are some clinically useful replacement measures such as homeostasis model assessment for insulin resistance (HOMA‐IR), quantitative insulin‐sensitivity check index (QUICKI) for assessment of insulin resistance (Freeman & Pennings, 2020). Result of a systematic review and meta‐analyses advise that those with diabetes mellitus must be encouraged to have sufficient intake of vegetables, nuts, fruits, and whole grains that are a subset of plant‐based diets and excellent sources of dietary fiber. Dietary fiber has a beneficial effect on insulin resistance (Reynolds et al., 2020). Also, plant‐based diets have been associated with much lower rates of obesity, hypertension, hyperlipidemia, cardiovascular mortality, and cancer (Dinu et al., 2017). Some clinical trials using vegetarian and vegan diets have shown significant improvements in body weight (Turner‐McGrievy et al., 2007) glycemic control (Barnard et al., 2006), and cardiovascular risk factors as compared with conventional therapeutic approaches (Jenkins et al., 2006).

Several studies have demonstrated that a diet rich in fruit and vegetables is related to increased bone mineral density (BMD) and improvement of bone microarchitecture (Kitchin & Morgan, 2007; New et al., 2000). Despite the positive influences of a diet rich in fruit and vegetables on bone health, it is unclear whether a plant‐based diet alone is enough for preventing adverse bone metabolic changes that may affect the development of osteoporosis (Diehl, 1998). A clinical trial showed that a plant‐based diet decrease calcium and vitamin D intake and cause an increase in N telopeptide biomarker that consistent with increased bone resorption (Merrill & Aldana, 2009).

The previous study on the association between plant‐based diet and risk of osteoporosis, inflammation and insulin resistance has been done in Western nations, and limited data are available in Middle‐East countries. People in the Middle East have different dietary patterns than those in Western countries.

Given the lack of information about the association between a plant‐based diet with osteoporosis, inflammation, and insulin resistance between Iranian older adults, we aimed to investigate the association between plant‐based diet and risk of osteoporosis, inflammation, and insulin resistance in this study.

## 2 MATERIALS AND METHODS

### 2.1 Participants

This cross‐sectional study was conducted on 178 elderly subjects (51 men and 127 women) with a mean age of 67.04 (60–83), referred to health centers in Tehran. The sample size of 160 was calculated using this formula: *n* = (pq $z^{2}$)/$E^{2}$ considering where *n* = sample size; $z^{2}$ = square of the confidence level in standard error units (1.96); p = the estimate of the proportion of older people; q = 1–p, or the estimated proportion of young people; and $E^{2}$ = the square of the maximum allowance for error between the true proportion and the sample proportion (=0.04). To compensate for the potential exclusion of participants due to under‐ and over‐reporting of total energy intake, or attrition due to other reasons, the final sample size of 178 participants was selected for inclusion. Participants were selected using a two‐stage cluster sampling of 25 Health centers in Tehran. Health centers in Tehran were divided into five regions: North, South, East, West, and Central and then prepared a list of health centers in each area, and 25 health centers were selected randomly (in attention to constraints budget and time) based on the number of health centers in each region proration. Then, the total number of samples (178) divided by the number of health centers (25) and obtained the number of samples in each home centers.

This study was conducted according to the guidelines laid down in the Declaration of Helsinki and all procedures involving research study participants were approved by the ethics committee of Tehran University of Medical Sciences (Ethics Number: IR.TUMS.VCR.REC.1396.2307). Written informed consent was obtained from all subjects/patients.

### 2.2 Anthropometric measurements

The patient's height was measured without shoes by a wall stadiometer with a sensitivity of 0.1 cm (Seca, Germany) and weight by digital scale (Seca 808, Germany) with an accuracy of 0.1 kg with light clothes (without a coat and rain coat). BMI was calculated by dividing weight in kilograms by the square of height in meters. Waist circumference was measured with a tape measure between the iliac crest and the lowest rib on the exhale. Body fat (%) was measured using a body composition analyzer (InBody 720, Biospace), where all participants were asked to follow these conditions before measurement: no food ingestion for at least 4 hr, minimal intake of 2 liters of water the day before, no physical activity for at least 8 hr, no coffee or alcoholic beverage consumption during at least 12 hr, and no diuretic use for at least 24 hr, prior to assessment, respectively. Participants were required to urinate immediately before the body composition test (Korth et al., 2007).

### 2.3 Assessment of other variables

We obtained information on age, sex, physical activity, smoking, marital status, diabetes, hypertension, and dyslipidemia through questionnaires. Smoking classified as a nonsmoker and former/current smoker. Diabetes, hypertension, and dyslipidemia quantified as yes or no. Physical activity was assessed using a validated short form of the International Physical Activity Questionnaire (IPAQ) (Moghaddam et al., 2012). Accordingly, subjects were grouped into three categories including very low (<600 MET‐minute/week), low (600–3000 MET‐minute/week), moderate, and high (>3,000 MET‐minute/week) (Wareham et al., 2003).

### 2.4 Laboratory investigation

10 ml of blood and 3 ml urine samples were obtained between the hours of 7–10 a.m. from all of the fasted participants. Then, blood sample was collected in acid‐washed test tubes without anticoagulant. After storing at room temperature for 30 min and clot formation, blood samples were centrifuged at 1,500 g for 20 min. Serums were stored at ‐ 80°C until future testing. Serum Human N‐MID Osteocalcin was measured by ELISA kit (Bioassay Technology Laboratory, Shanghai Crystal Day Biotech Co., Ltd.), with CV < 10% and sensitivity of 0.22 ng/ml. The Human C‐telopeptide of type Ⅰ collagen (u‐CTX‐I) ELISA kit (Bioassay Technology Laboratory, Shanghai Crystal Day Biotech Co., Ltd.), with intra‐Assay: CV < 10% and sensitivity of 0.24 ng/ml was used for the quantitative measurement of CTX‐I in urine. The measurement of highly sensitive C‐reactive protein (hs‐CRP) was performed by immunoturbidimetric assay based on the kit instructions (Pars Azmoon, Iran, and Tehran). The 25(OH) D and PTH were assessed using an enzymatic method, using commercial kits (with Pars Azmoon, Iran, Tehran and DRG), respectively. HOMA‐IR was calculated using the formula: HOMA‐IR = [glucose (nmol/L) * insulin (µU/ml)/22.5], using fasting values (Matthews et al., 1985), and the QUICKI was calculated using the formula: 1 / (log (fasting insulin μU/ml) + log (fasting glucose mg/dl)) (Katz et al., 2000).

### 2.5 Dietary assessment

Dietary intakes of subjects were evaluated using a valid and reliable semi‐quantitative food frequency questionnaire (FFQ), with 168 food items (Esfahani et al., 2010). Trained researchers via face‐to‐face interviews asked subjects to report their frequency of intake of each food item, during the past year on a daily, weekly, or monthly basis. These reports were converted to daily intakes, and then, we used this dietary data to generate three versions of a plant‐based diet: an overall PDI, hPDI, and uPDI (Satija et al., 2016). Table S1 details examples of food group constituents. We created 18 food groups based on nutrient and culinary similarities within the larger categories of healthy plant foods (whole grains, fruits, vegetables, nuts, legumes, vegetable oils, tea/coffee), less healthy plant foods (fruit juices, refined grains, potatoes, sugar‐sweetened beverages, sweets/desserts), and animal foods (animal fat, dairy, eggs, fish/seafood, meat, miscellaneous animal‐based foods). The classification of mixed composition foods was according to the predominate ingredient. Participants were ranked into quintiles according to their food intakes, which were subsequently given positive or inverse scores. With positive scores, participants above the highest quintile of a food group received a score of 5 and those below the lowest quintile received a score of 1. With inverse scores, this pattern of scoring was inversed. For PDI, plant food groups were given positive scores, while animal food groups were given inverse scores. For hPDI, positive scores were allocated to healthy plant food groups, and inverse scores to less healthy plant food groups and animal food groups. Finally, for uPDI, positive scores were allocated to less healthy plant food groups, and inverse scores to healthy plant food groups and animal food groups. The 18 food group scores were summed to establish the indices.

### 2.6 Statistical analysis

All statistical analysis was performed with the SPSS (Statistical Package for Social Sciences) for Windows 25.0 software package (SPSS). The level of statistical significance was pre‐set at *p* < .05. The normality of data was evaluated by the Kolmogorov and Smirnov test. People were grouped based on the tertiles of PDI, hPDI, and uPDI. To compare general characteristics among tertiles, we used one‐way ANOVA for quantitative variables also qualitative variables were evaluated by chi‐square tests. Pearson correlation was conducted to assess the relation of food groups intake with test variables including serum osteocalcin, urine CTX‐I, hs‐CRP, 25(OH) D, PTH, serum insulin, HOMA‐IR, and QUICKI. Multivariate‐adjusted means were performed to evaluate the relationship between tertiles of PDI, uPDI, and hPDI with other variables including osteocalcin, urine CTX‐I, hs‐CRP, 25(OH) D, PTH, serum insulin, HOMA‐IR, and QUICKI (adjusted for age, sex, BMI, smoking, physical activity, marital status, disease, and energy intake). Multiple linear regression analysis was used to evaluate the association between serum osteocalcin, urine CTX‐I, hs‐CRP, 25(OH) D, PTH, serum insulin, HOMA‐IR, and QUICKI with PDI, uPDI, and hPDI score after adjustment for covariates, including age, sex, BMI, smoking, physical activity, marital status, disease, and energy intake. In all the above‐mentioned analyses, the first tertile was regarded as the reference category.

## 3 RESULTS

General characteristics of study participants across categories of PDI, hPDI, and uPDI are presented in Table 1. There were no significant differences across tertiles plant‐based diet indexes in terms of the mean of age, weight, BMI, WHR, WC, percent of body fat, and distribution of sex, physical activity, smoking status, marital status, diabetes, hypertension, and dyslipidemia.

**TABLE 1:** Characteristics of study participants by tertiles of plant‐based diet indexes

	Plant‐based Diet Indexes			*p*
	PDI			
	T1	T2	T3	
Mean score	43.55 ± 3.138	51.17 ± 2.14	58.83 ± 3.567	
Age (year)	67.22 ± 5.18	66.97 ± 6.82	66.94 ± 6.63	.96
Weight (kg)	70.68 ± 10.75	72.81 ± 13.53	74.06 ± 13.61	.38
BMI (kg/m^2^)	29.59 ± 4.08	29.49 ± 4.80	30.44 ± 5.55	.51
WHR	0.94 ± 0.07	0.96 ± 0.07	0.93 ± 0.10	.37
WC (cm)	98.27 ± 9.32	100.02 ± 11.02	100.17 ± 11.01	.56
Body fat (%)	43.27 ± 7.11	41.19 ± 7.46	44.38 ± 9.01	.07
Sex				
Male	13 (25.5%)	25 (49%)	13 (25.5%)	.24
Female	42 (33.1%)	45 (35.4%)	40 (31.5%)	
Physical activity				
Very low	28 (34.1%)	31 (37.8%)	23 (28%)	.53
Low	15 (24.6%)	29 (47.5%)	17 (27.9%)	
Moderate and upper	11 (34.4%)	10 (31.3%)	11 (34.4%)	
Smoking				
Not smoking	47 (31.5%)	55 (36.9%)	47 (31.5%)	.29
Former and current smoking	8 (27.6%)	15 (51.7%)	6 (20.7%)	
Marital status, married, *n* (%)	35 (20.0%)	49 (28.0%)	38 (21.7%)	.77
Diabetes, yes, *n* (%)	23 (12.9%)	29 (16.3%)	18 (39.3%)	.63
Hypertension, yes, *n* (%)	27 (15.2%)	43 (24.2%)	29 (55.6%)	.38
Dyslipidemia,yes, *n* (%)	34 (19.1%)	39 (21.9%)	20 (11.2%)	.03
**hPDI**				
Mean score	44 ± 3.394	51.07 ± 1.472	57.38 ± 3.383	
Age (year)	66.60 ± 5.61	66.78 ± 5.99	67.66 ± 6.58	.59
Weight (kg)	73.20 ± 13.08	73.01 ± 13.43	71.49 ± 11.96	.72
BMI (kg/m^2^)	29.51 ± 5.38	30.09 ± 4.42	29.79 ± 4.73	.81
WHR	0.95 ± 0.08	0.95 ± 0.08	0.94 ± 0.09	.71
WC (cm)	100.10 ± 10.74	100.38 ± 10.64	98.25 ± 10.19	.47
Body fat (%)	42.97 ± 8.39	42.76 ± 7.17	42.64 ± 8.29	.97
Sex				
Male	14 (27.5%)	17 (33.3%)	20 (39.2%)	.78
Female	41 (32.3%)	42 (33.1%)	44 (34.6%)	
Physical activity				
Very low	25 (32.9%)	27 (30.5%)	30 (36.6%)	.87
Low	18 (29.5%)	21 (34.4%)	22 (36.1%)	
Moderate and upper	8 (25%)	13 (40.6%)	11 (34.4%)	
Smoking				
Not smoking	46 (30.9%)	49 (32.9%)	54 (36.2%)	.98
Former and current smoking	9 (31%)	10 (34.5%)	10 (34.5%)	
Marital status, married, *n* (%)	38 (21.7%)	43 (24.6%)	41 (23.4%)	.67
Diabetes, yes, *n* (%)	19 (10.7%)	24 (13.5%)	27 (15.2%)	.67
Hypertension, yes, *n* (%)	30 (16.9%)	32 (18.0%)	37 (20.8%)	.90
Dyslipidemia, yes, *n* (%)	29 (16.3%)	32 (18.0%)	32 (18.0%)	.89
**uPDI**				
Mean score	45.78 ± 3.36	54.32 ± 2.17	62.14 ± 3.96	
Age (year)	66.60 ± 5.15	67.77 ± 7.06	66.64 ± 5.74	.48
Weight (kg)	71.27 ± 12.68	71.58 ± 13.35	74.90 ± 12.03	.24
BMI (kg/m^2^)	29.51 ± 4.54	29.66 ± 4.85	30.27 ± 5.11	.67
WHR	0.95 ± 0.07	0.94 ± 0.09	0.94 ± 0.08	.61
WC (cm)	98.94 ± 10.59	99.13 ± 10.89	100.58 ± 10.04	.66
Body fat (%)	115.93 ± 42.66	105.75 ± 34.92	110.32 ± 48.96	.42
Sex				
Male	18 (35.3%)	19 (37.3%)	14 (27.5%)	.73
Female	39 (30.7%)	46 (36.2%)	42 (33.1%)	
Physical activity				
Very low	29 (35.4%)	26 (31.7%)	27 (32.9%)	.65
Low	19 (31.1%)	23 (37.7%)	19 (31.1%)	
Moderate and upper	8 (25%)	15 (46.9%)	19 (28.1%)	
Smoking				
Not smoking	48 (32.4%)	53 (35.8%)	47 (31.8%)	.97
Former and current smoking	9 (31%)	11 (37.9%)	9 (31%)	
Marital status, married, *n* (%)	41 (23.4%)	41 (23.4%)	40 (22.9%)	.39
Diabetes, yes, *n* (%)	23 (12.9%)	24 (13.5%)	23 (12.9%)	.88
Hypertension, yes, *n* (%)	34 (19.1%)	33 (18.5%)	32 (18.0%)	.52
Dyslipidemia, yes, *n* (%)	34 (19.1%)	26 (14.6%)	33 (18.5%)	.04

Note

*p* value less than .05 was considered significant.

Values are based on mean ± standard deviation or frequency (reported percentage).

One‐way ANOVA for quantitative data and chi‐square test for qualitative data have been used.

Abbreviations: BMI, body mass index; WC, waist circumference; WHR, waist to hip ratio.

Dietary intakes of study participants across categories of PDI, hPDI, and uPDI are presented in Table 2. Participants with the highest PDI score had significantly higher whole grains (P_trend_ = 0.004), fruits (P_trend_ = 0.001), legumes (P_trend_ = 0.003), vegetable oils (P_trend_ = 0.006), tea & coffee (P_trend_ = 0.01), fruit juices (P_trend_ = 0.02), refined grains (P_trend_ = 0.01), potatoes (P_trend_ = 0.04), and sugar‐sweetened beverages (P_trend_ = 0.03). After adjustment for confounders, the association remained unchanged. Participants with the highest hPDI score had significantly lower vegetable oils (P_trend_ = 0.04), refined grains (P_trend_ < 0.001), potatoes (P_trend_ = 0.01), sugar‐sweetened beverages (P_trend_ < 0.001), meat (P_trend_ < 0.001), and misc. animal‐based foods (P_trend_ = 0.02). After adjustment for confounders, the association remained unchanged. Participants with the highest uPDI score had significantly lower whole grains (P_trend_ = 0.01), fruits (P_trend_ = 0.003), vegetables (P_trend_ = 0.002), nuts (P_trend_ = 0.001) animal fat (P_trend_ = 0.002), dairy (P_trend_ < 0.001), egg (P_trend_ = 0.003), fish or seafood (P_trend_ = 0.005), meat (P_trend_ = 0.002), and misc. animal‐based foods (P_trend_ = 0.03). After adjustment for confounders, the association remained unchanged. Participants with the highest uPDI score had significantly higher sugar‐sweetened (P_trend_ < 0.001) and also after adjustment for confounders the association remained unchanged.

**TABLE 2:** Dietary intakes according to tertile of plant‐based diet indices^†^

	Mean ± *SD*	PDI			*p* *	*p* ^†^
		Tertile 1	Tertile 2	Tertile 3		
Whole grains (g/day)	183.40 ± 157.47	144.41 ± 108.81	179.17 ± 153.33	229.46 ± 192.67	.004	.01
Fruits (g/day)	499.02 ± 274.00	377.32 ± 224.99	537.19 ± 263.44	574.92 ± 295.63	.001	.001
Vegetables (g/day)	460.16 ± 215.65	412.40 ± 475.83	475.83 ± 214.05	489.04 ± 219.52	.17	.45
Nuts (g/day)	16.70 ± 27.58	15.45 ± 36.44	13.04 ± 13.70	22.83 ± 30.04	.10	.27
Legumes (g/day)	32.05 ± 24.64	23.83 ± 27.29	33.91 ± 22.93	38.11 ± 21.97	.003	<.001
Vegetable oils (g/day)	18.52 ± 13.20	14.56 ± 10.34	17.12 ± 11.61	24.46 ± 15.57	.006	.02
Tea & Coffee (g/day)	646.44 ± 351.86	547.88 ± 301.72	622.78 ± 352.29	799.97 ± 364.67	.01	.01
Fruit juices (g/day)	27.28 ± 39.58	14.52 ± 28.94	23.52 ± 32.64	45.48 ± 50.22	.02	.04
Refined grains (g/day)	259.60 ± 152.94	201.54 ± 126.21	257.10 ± 161.05	323.16 ± 144.81	.01	.03
Potatoes (g/day)	22.04 ± 21.76	12.51 ± 10.36	22.01 ± 22.30	31.95 ± 25.38	.04	<.001
Sugar sweetened beverages (g/day)	29.29 ± 58.15	16.30 ± 55.60	21.35 ± 30.50	53.27 ± 78.85	.03	<.001
Sweets and Desserts (g/day)	44.56 ± 136.31	18.73 ± 20.01	62.45 ± 213.88	47.75 ± 31.37	.23	.55
Animal fat (g/day)	4.37 ± 7.80	3.10 ± 5.62	5.14 ± 8.69	4.67 ± 8.47	.82	.72
Dairy (g/day)	384.40 ± 225.34	327.38 ± 198.80	412.70 ± 237.55	406.19 ± 227.85	.49	.08
Egg (g/day)	13.78 ± 13.92	15.19 ± 12.20	13.80 ± 11.85	12.27 ± 17.72	.59	.42
Fish or Seafood (g/day)	9.65 ± 10.49	11.18 ± 10.25	10.39 ± 12.13	7.09 ± 7.75	.06	.04
Meat (g/day)	50.70 ± 37.20	48.38 ± 24.23	56.54 ± 45.14	45.41 ± 36.27	.52	.74
Misc. Animal‐based foods (g/day)	7.32 ± 9.58	6.08 ± 4.81	6.33 ± 5.76	9.91 ± 15.32	.06	.03

	Mean ± *SD*	hPDI			*p* *	*p* ^†^
		Tertile 1	Tertile 2	Tertile 3		
Whole grains (g/day)	183.40 ± 157.47	148.24 ± 162.65	187.50 ± 148.05	209.84 ± 158.14	.02	.01
Fruits (g/day)	499.02 ± 274.00	430.08 ± 226.83	529.06 ± 325.69	530.59 ± 251.33	.09	.03
Vegetables (g/day)	460.16 ± 215.65	415.27 ± 180.79	464.95 ± 220.89	494.32 ± 234.00	.23	.02
Nuts (g/day)	16.70 ± 27.58	12.28 ± 17.29	23.75 ± 40.76	14.00 ± 16.72	.21	.82
Legumes (g/day)	32.05 ± 24.64	32.82 ± 23.47	29.00 ± 20.08	34.20 ± 29.14	.70	.58
Vegetable oils (g/day)	18.52 ± 13.20	22.41 ± 13.50	17.78 ± 11.83	15.85 ± 13.55	.04	<.001
Tea & Coffee (g/day)	646.44 ± 351.86	639.53 ± 371.38	629.07 ± 325.04	668.39 ± 362.67	.47	.65
Fruit juices (g/day)	27.28 ± 39.58	33.44 ± 42.09	27.36 ± 44.18	21.92 ± 32.01	.30	.12
Refined grains (g/day)	259.60 ± 152.94	324.37 ± 134.91	252.87 ± 113.26	210.14 ± 179.20	<.001	<.001
Potatoes (g/day)	22.04 ± 21.76	27.79 ± 25.05	22.27 ± 16.99	16.88 ± 21.69	.01	.01
Sugar sweetened beverages (g/day)	29.29 ± 58.15	47.41 ± 64.13	35.72 ± 72.71	7.79 ± 17.73	<.001	<.001
Sweets and Desserts (g/day)	44.56 ± 136.31	49.44 ± 30.66	65.73 ± 233.13	20.87 ± 18.75	.22	.25
Animal fat (g/day)	4.37 ± 7.80	5.48 ± 8.32	3.75 ± 6.1	3.99 ± 8.65	.27	.41
Dairy (g/day)	384.40 ± 225.34	398.56 ± 232.01	374.29 ± 223.65	381.55 ± 224.03	.30	.91
Egg (g/day)	13.78 ± 13.92	14.96 ± 11.06	15.07 ± 18.23	11.56 ± 11.22	.42	.15
Fish or Seafood (g/day)	9.65 ± 10.49	11.88 ± 10.83	9.29 ± 10.12	8.07 ± 10.35	.16	.04
Meat (g/day)	50.70 ± 37.20	64.06 ± 48.39	50.25 ± 32.99	39.64 ± 24.47	<.001	.01
Misc. Animal‐based foods (g/day)	7.32 ± 9.58	9.20 ± 8.77	8.62 ± 13.60	4.51 ± 2.98	.02	<.001

	Mean ± *SD*	uPDI			*p* *	*p* ^†^
		Tertile 1	Tertile 2	Tertile 3		
Whole grains (g/day)	183.40 ± 157.47	220.26 ± 180.81	207.66 ± 163.52	117.94.39	.01	.001
Fruits (g/day)	499.02 ± 274.00	614.13 ± 232.82	500.90 ± 274.75	379.69 ± 265.23	.003	.02
Vegetables (g/day)	460.16 ± 215.65	579.55 ± 213.64	448.97 ± 211.70	351.64 ± 155.35	.002	<.001
Nuts (g/day)	16.70 ± 27.58	21.97 ± 24.87	14.68 ± 20.68	13.68 ± 35.73	.001	<.001
Legumes (g/day)	32.05 ± 24.64	38.84 ± 27.19	32.41 ± 24.45	24.71 ± 20.08	.002	.34
Vegetable oils (g/day)	18.52 ± 13.20	19.46 ± 11.30	20.72 ± 15.04	14.99 ± 12.17	.03	.09
Tea & Coffee (g/day)	646.44 ± 351.86	674.83 ± 364.46	670.89 ± 328.76	589.16 ± 364.04	.44	.38
Fruit juices (g/day)	27.28 ± 39.58	29.13 ± 47.32	22.74 ± 29.74	30.67 ± 41.15	.23	.79
Refined grains (g/day)	259.60 ± 152.94	239.03 ± 125.23	266.42 ± 173.70	272.62 ± 153.40	.41	.25
Potatoes (g/day)	22.04 ± 21.76	17.56 ± 15.85	21.43 ± 14.47	27.30 ± 31.22	.07	.03
Sugar sweetened beverages (g/day)	29.29 ± 58.15	11.76 ± 32.03	29.03 ± 34.17	47.44 ± 88.47	<.001	.001
Sweets and Desserts (g/day)	44.56 ± 136.31	32.92 ± 30.57	34.11 ± 25.16	68.55 ± 239.27	.38	.22
Animal fat (g/day)	4.37 ± 7.80	7.55 ± 10.16	3.35 ± 7.14	2.32 ± 3.93	.002	<.001
Dairy (g/day)	384.40 ± 225.34	477.77 ± 270.18	386.03 ± 207.17	287.47 ± 143.32	<.001	<.001
Egg (g/day)	13.78 ± 13.92	19.86 ± 17.99	12.11 ± 9.44	9.52 ± 11.42	.003	.001
Fish or Seafood (g/day)	9.65 ± 10.49	13.68 ± 11.95	10.22 ± 10.83	4.89 ± 5.64	.005	.02
Meat (g/day)	50.70 ± 37.20	63.67 ± 44.29	49.06 ± 31.46	39.41 ± 31.57	.002	.001
Misc. Animal‐based foods (g/day)	7.32 ± 9.58	9.99 ± 13.91	7.13 ± 7.98	4.82 ± 3.26	.03	.03

Note

*p* value less than .05 was considered significant.

Values are based on mean ± standard deviation

Abbreviations: hPDI, healthy plant‐based diet index; PDI, plant‐based diet index; uPDI, unhealthy plant‐based diet index.

* *p* for trend calculated by analysis of variance (ANOVA).

^†^ *p* for trend adjusted for total energy intake.

Dietary groups of vegetables (*r *= .15, *p* = .03), nuts (*r *= .16, *p* = .03), dairy (*r *= .25, *p* = .001), eggs (*r *= .27, *p* < .001), red meat, and animal products (*r *= .25, *p* = .001) were directly correlated with osteocalcin. Consumption of vegetables was also inversely associated with hs‐CRP serum levels (*r* = −.30, *p* < .001). Consumption of refined grains was negatively associated with PTH serum levels (*r* = −.16, *p* = .03). Refined grains were also had a positive association with serum insulin concentration (*r *= .14, *p* = .04). Potatoes consumption had a positive association with serum insulin concentration (*r *= .15, *p* = .04) (Table 3.) Participants with the highest PDI score had significantly lower PTH (P_trend_ = 0.02) just after adjusting for confounders, including age, sex, smoking, physical activity, marital status, income levels, BMI, total energy, diabetes, hypertension, and dyslipidemia (P_trend_ = 0.03), and they also had significantly higher insulin serum levels (P_trend_ = 0.03), which was also seen after the confounders were adjusted (P_trend_ = 0.02). There was only a significant association between the insulin sensitivity index with people who were in the highest hPDI score, after adjusting the confounders (P_trend_ = 0.03). Participants with the highest uPDI score had significant lower osteocalcin (P_trend_ < 0.001), PTH (P_trend_ = 0.04), and they had significantly higher insulin serum levels (P_trend_ = 0.01). After adjustment for confounders, the association remained unchanged for both osteocalcin (P_trend_ = 0.01), PTH (P_trend_ = 0.02) and insulin (P_trend_ = 0.03). Participants with the highest uPDI score had significantly higher insulin resistance (P_trend_ = 0.05) but after adjusting for confounders, the association was not significant (P_trend_ = 0.09) (Table 4). According to Multiple regression analysis models shown in Table 5, PTH levels are inversely associated with PDI score. This significance did not change with the adjustment of the confounders (β=−0.18, *p* = .01). Also, serum insulin concentration was negatively associated with PDI score (β=−0.10, *p* = .04). Urine CTX‐1 levels were significantly associated with hPDI score (β=−0.06, *p* = .04). u‐CTX‐1 levels are inversely associated with uPDI score. This significance did not change with the adjustment of the confounders (β=−0.28, *p* < .001). Adherence to an uPDI significantly increases insulin serum levels even after adjusting for confounders (β = 0.16, *p* = .04), but there was no significant linear association between insulin sensitivity markers such as HOMA_IR and Quicki with PDI, hPDI, and uPDI.

**TABLE 3:** Correlation of food groups intake with biochemical markers of bone, inflammation, and insulin

Subgroup analysis	Osteocalcin		Urine CTX‐I		25(OH)D		PTH		hs‐CRP		Insulin		HOMA_IR		Quicki	
	*r*	*p*	*r*	*p*	*r*	*p*	*r*	*p*	*r*	*p*	*r*	*p*	*r*	*p*	*r*	*p*
Whole grains (g/day)	.08	.24	.11	.12	−.09	.20	.08	.26	−.04	.55	−.13	.07	−.11	.13	.03	.65
Fruits (g/day)	.01	.79	−.02	.78	−.04	.55	−.50	.50	−.05	.45	−.01	.94	.04	.52	.01	.81
Vegetables (g/day)	.15	.03	−.03	.69	.01	.87	−.01	.85	−.30	<.001	−.4	.58	−.01	.94	.04	.57
Nuts (g/day)	.16	.03	.06	.37	.04	.55	−.08	.24	.01	.86	.02	.78	−.02	.79	−.01	.87
Legumes (g/day)	.03	.62	−.06	.42	−.06	.39	−.06	.45	.01	.92	.01	.81	.12	.11	−.05	.44
Vegetable oils (g/day)	.07	.33	−.01	.84	−.05	.45	.02	.77	.03	.62	.01	.89	−.02	.77	−.08	.29
Tea & Coffee (g/day)	.07	.34	−.03	.63	−.04	.59	−.03	.64	−.07	.33	.02	.79	−.04	.56	.01	.88
Fruit juices ( g/day )	.25	.001	.04	.51	−.04	.58	−.08	.24	−.06	.35	−.02	.77	−.02	.75	.06	.40
Refined grains ( g/day )	.05	.45	−.01	.85	−.05	.49	−.16	.03	.02	.79	.14	.04	.03	.74	−.04	.59
Potatoes ( g/day )	−.12	.08	−.06	.41	−.08	.26	.03	.62	.12	.09	.15	.04	.08	.28	−.11	.15
Sugar sweetened beverages ( g/day )	−.07	.29	−.03	.67	−.06	.38	−.01	.98	−.02	.76	.07	.34	.02	.73	−.10	.17
Sweets and Desserts ( g/day )	−.02	.73	−.05	.46	−.07	.32	.03	.61	.01	.91	−.05	.46	−.04	.60	.03	.66
Animal fat ( g/day )	.08	.26	.02	.72	−.07	.36	.13	.07	.02	.76	−.09	.23	−.07	.31	.05	.49
Dairy ( g/day )	.25	.001	−.02	.98	.06	.39	.02	.72	−.01	.80	.01	.89	.01	.89	−.02	.71
Egg ( g/day )	.27	<.001	−.04	.57	.05	.50	−.10	.18	−.06	.43	.01	.83	−.03	.64	−.03	.67
Fish or Seafood ( g/day )	−.03	.61	−.04	.58	.06	.36	−.01	.93	−.03	.67	−.13	.07	−.12	.09	.10	.16
Meat ( g/day )	.15	.04	.03	.61	−.04	.51	.04	.56	−.03	.62	−.01	.89	−.02	.75	.01	.88
Misc. Animal‐based foods ( g/day )	.25	.001	.12	.10	.03	.67	.04	.59	.07	.32	−.01	.86	−.02	.78	−.05	.51

Note

Data are Pearson correlation coefficients.

Abbreviations: 25(OH)D, 25‐hydroxyvitamin D; HOMA‐IR, Homeostasis Model Assessment for Insulin Resistance; hs‐CRP, High sensitivity c‐reactive protein; PTH, parathormone; QUICKI, Quantitative Insulin‐sensitivity Check Index; u‐CTx, urine terminal telopeptide of type I collagen.

**TABLE 4:** Multivariate adjusted means for biochemical markers of bone, inflammation, and insulin across tertiles of plant‐based diet indices

	All Mean ± *SD*	Tertiles of PDI			*p* *	*p* ^†^
		Tertile1	Tertile2	Tertile3		
Osteocalcin (ng/ml)	20.5 ± 14.5	21.6 ± 15.0	19.9 ± 14.3	20.0 ± 14.5	.57	.51
uCTX‐I (ng/ml)	32.5 ± 7.30	32.7 ± 8.65	31.7 ± 7.08	32.5 ± 7.30	.89	.26
PTH (ng/L)	50.7 ± 27.1	56.6 ± 34.3	51.6 ± 26.9	45.1 ± 18.6	.02	.03
hs‐CRP (mg/L)	4.88 ± 13.9	3.84 ± 3.88	7.54 ± 23.4	3.27 ± 3.24	.82	.57
25(OH)D (ng/ml)	11.0 ± 6.77	11.4 ± 6.40	10.5 ± 6.57	11.0 ± 7.30	.70	.88
HOMA_IR	3.78 ± 5.48	3.88 ± 7.26	2.67 ± 2.32	4.75 ± 5.69	.39	.22
Insulin (µlu/ml)	13.6 ± 14.5	12.1 ± 11.6	10.4 ± 9.81	18.0 ± 19.1	.03	.02
Quicki	0.34 ± 0.04	0.34 ± 0.04	0.35 ± 0.04	0.33 ± 0.04	.16	.45

	All Mean ± *SD*	Tertiles of hPDI			*p* *	*p* ^†^
		Tertile1	Tertile2	Tertile3		
Osteocalcin (ng/ml)	20.5 ± 14.5	21.7 ± 16.0	20.7 ± 16.6	19.1 ± 10.4	.33	.72
uCTX‐I (ng/ml)	32.3 ± 7.64	31.7 ± 7.01	32.0 ± 8.65	33.1 ± 7.26	.30	.33
PTH (ng/L)	50.7 ± 27.1	49.6 ± 21.5	53.1 ± 27.7	49.5 ± 31.4	.98	.65
hs‐CRP (mg/L)	4.88 ± 13.9	4.91 ± 9.74	5.34 ± 21.8	4.41 ± 4.50	.84	.54
25(OH)D (ng/ml)	11.0 ± 6.77	10.9 ± 6.46	11.1 ± 7.30	10.9 ± 6.64	.94	.86
HOMA_IR	3.78 ± 5.48	3.97 ± 3.94	3.18 ± 4.88	4.19 ± 7.21	.83	.57
Insulin (µlu/ml)	13.6 ± 14.5	15.2 ± 16.2	12.5 ± 15.3	13.1 ± 11.9	.43	.39
Quicki	0.34 ± 0.04	0.33 ± 0.04	0.35 ± 0.04	0.33 ± 0.04	.92	.03

	All Mean ± *SD*	Tertiles of uPDI			*p* *	*p* ^†^
		Tertile1	Tertile2	Tertile3		
Osteocalcin (ng/ml)	20.5 ± 14.5	27.5 ± 21.0	17.8 ± 9.50	16.5 ± 7.02	<.001	.01
uCTX‐I (ng/ml)	32.3 ± 7.64	32.0 ± 8.28	33.5 ± 7.83	31.2 ± 6.64	.58	.39
PTH (ng/L)	50.7 ± 27.1	58.9 ± 36.4	43.9 ± 19.0	50.5 ± 22.1	.04	.02
hs‐CRP (mg/L)	4.88 ± 13.9	6.15 ± 22.2	5.00 ± 9.54	3.50 ± 3.81	.32	.58
25(OH)D (ng/ml)	11.0 ± 6.77	10.4 ± 6.48	12.0 ± 7.02	10.4 ± 6.74	.94	.79
HOMA_IR	3.78 ± 5.48	3.14 ± 4.51	3.16 ± 2.91	5.18 ± 8.01	.05	.12
Insulin (µlu/ml)	13.6 ± 14.5	10.7 ± 11.3	12.5 ± 12.0	17.9 ± 18.9	.01	.03
Quicki	0.34 ± 0.04	0.34 ± 0.04	0.34 ± 0.04	0.33 ± 0.04	.24	.76

*p* value less than .05 was considered significant.

Values are based on mean ± standard deviation.

Abbreviations: µlu/ml, micro international unit per milliliter; HOMA_IR, Homeostasis Model Assessment for Insulin Resistance; hs‐CRP, high sensitivity c‐reactive protein; mg/L, milligram per liter; ng/L, nanogram per liter; ng/mL, nanogram per milliliter; OH, hydroxyl;PTH, parathormone; QUICKI, Quantitative Insulin‐sensitivity Check Index; u‐CTx, urinary C‐terminal telopeptide of type I collagen.

* P for trend.

^†^ P for trend adjusted for age, sex, smoking, marital status, Disease, physical activity, BMI and energy.

**TABLE 5:** Multiple regression analysis models exploring the association of biochemical markers of bone, inflammation, and insulin with plant‐based diet indices

	PDI			hPDI			uPDI		
	β ± SE	95% CI	*p*_value_	β ± SE	95% CI	*p*_value_	β ± SE	95% CI	*p*_value_
Osteocalcin									
Model 1	−0.08 ± 0.16	−0.51,0.13	.25	−0.05 ± 0.18	−0.49,0.22	.45	−0.30 ± 0.14	−0.87,−0.30	<.001
Model 2	−0.10,0.17	−0.56,0.10	.17	−0.03 ± 0.18	−0.43,0.30	.73	−0.28 ± 0.15	−0.84,−0.26	<.001
Urine CTX‐I									
Model 1	−0.05 ± 0.08	−0.24,0.10	.43	−0.02 ± 0.09	−0.16,−0.21	.02	0.01 ± 0.08	−0.14,0.17	.85
Model 2	−0.04 ± 0.09	−0.22,0.12	.58	−0.06 ± 0.09	−0.13,−0.27	.04	0.03 ± 0.08	−0.12,0.19	.65
25(OH)D									
Model 1	−0.08 ± 0.07	−0.23,0.06	.28	−0.01 ± 0.08	−0.14,0.18	.83	0.04 ± 0.07	−0.10,0.17	.60
Model 2	−0.07 ± 0.08	−0.23,0.08	.33	−0.04 ± 0.08	−0.22,0.11	.54	0.05 ± 0.07	−0.09,0.19	.48
PTH									
Model 1	−0.17 ± 0.30	−1.30,−0.10	.02	−0.04 ± 0.33	−0.87,0.46	.54	−0.13 ± 0.28	−1.04,0.05	.08
Model 2	−0.18 ± 0.31	−1.40,−0.15	.01	−0.02 ± 0.33	−0.79,0.57	.75	−0.14 ± 0.28	−1.10,0.02	.06
hs‐CRP									
Model 1	−0.03 ± 0.16	−0.40,0.24	.60	−0.01 ± 0.17	−0.39,0.28	.75	−0.05 ± 0.14	−0.38,0.19	.51
Model 2	−0.02 ± 0.16	−0.37,0.29	.81	−0.01 ± 0.18	−0.37,0.33	.91	−0.03 ± 0.15	−0.36,0.22	.63
Insulin									
Model 1	−0.11 ± 0.17	−0.09,−0.60	.02	−0.06 ± 0.18	−0.52,0.20	.38	0.15 ± 0.16	0.001,0.61	.04
Model 2	−0.10 ± 0.18	−0.13,−0.59	.04	−0.08 ± 0.19	−0.58,0.16	.27	0.16 ± 0.16	0.01,0.64	.04
HOMA_IR									
Model 1	0.06 ± 0.06	−0.07,0.18	.43	−0.03 ± 0.07	−0.10,−0.17	.03	0.08 ± 0.05	−0.05,0.17	.28
Model 2	0.07 ± 0.07	−0.07,0.19	.38	−0.02 ± 0.07	−0.12,−0.16	.04	0.08 ± 0.06	−0.05,0.18	.30
Quicki									
Model 1	−0.08 ± 0.001	−0.002,0.001	.30	0.02 ± 0.001	−0.001,0.001	.78	−0.07 ± 0.001	−0.001,0.001	.36
Model 2	−0.06 ± 0.001	−0.002,0.001	.40	0.03 ± 0.001	−0.001,0.001	.64	−0.08 ± 0.001	−0.001,0.001	.33

Note

*p* value less than 0.05 was considered significant. *p*_value_ obtained from Linear regression.

Model 1: Not adjusted for any variables.

Model2: The model was adjusted for age, sex, energy intake, marital status, smoking, disease, physical activity, and BMI.

Abbreviations: CI, confidence interval; HOMA_IR, Homeostasis Model Assessment for Insulin Resistance; hs‐CRP, high sensitivity c‐reactive protein; OH, hydroxyl; PTH, parathormone; QUICKI, Quantitative Insulin‐sensitivity Check Index; SE, standard error; u‐CTx, urinary C‐terminal telopeptide of type I collagen; β, standardized coefficients.

## 4 DISCUSSION

In this cross‐sectional study of Iranian older adults, we found no significant association between overall PDI and osteocalcin as a bone formation marker or uCTX‐1 as a resorption marker. These results as well as seen in hPDI but an inverse association observed between uPDI and osteocalcin. These findings confirm support to the findings of earlier intervention studies which also failed to show any significant effect of increased fruit and vegetable consumption on bone turnover markers (McTiernan et al., 2009; Nowson et al., 2009). Results from a 2‐year study in women, aged 55–65 years, showed no significant effect of increased fruit and vegetable consumption on bone turnover markers or BMD loss (Macdonald et al., 2008). Similarly, the Women's Health Initiative Dietary Modification Trial, which was also conducted in postmenopausal women, failed to show any significant effect of increased fruit and vegetable consumption (five or more servings per day) on vertebral or total body BMD or fracture over an eight‐year period (McTiernan et al., 2009). In contrast, a clinical trial study showing increased intake of a selection of vegetables/herbs and fruit decreased bone formation (P1NP) and resorptive (CTX) markers in postmenopausal women (Gunn et al., 2015).

No significant association observed between overall PDI, hPDI, and uPDI and hs‐CRP serum levels and in this study. A cross‐sectional study investigated that a higher intake of healthy plant foods, instead of unhealthy plant foods such as sweets and desserts, reduced hs‐CRP levels. Furthermore, higher adherence to uPDI (refined grains, starches sweetened with sugar, sweets, desserts, and juices) in women was not associated with a higher level of hs‐CRP (Bolori et al., 2019). In the Nurse's Health Study, adherence to a healthy vegetarian diet significantly reduced insulin resistance, inflammatory biomarkers in women participants (Chrysohoou et al., 2013). Several studies designed to evaluate the effect of Dietary Approaches to Stop Hypertension (DASH) diet on hs‐CRP, and other inflammatory markers (such as IL‐6 and TNF‐α) have shown conflicting results. Some RCTs have supported the link between DASH diet adherence and reduced CRP levels, while the others did not reveal the same results (Asemi & Esmaillzadeh, 2015; Erlinger et al., 2003; King et al., 2007; Roussell et al., 2012). The DASH diet emphasizes the increasing intake of fruit, vegetables, whole grains, low‐fat dairy products, fish, poultry, and nuts and a reduced intake of fats, red meat, and sugar‐containing beverages. Furthermore, this diet emphasizes low intake of total and saturated fat as well as cholesterol and increased consumption of magnesium, potassium, calcium, fiber, and protein (Appel et al., 1997). The conflicting results can be attributed to differences in study design and its sample size as well as differences in the dietary composition of plant‐based dietary pattern.

In the present study, no significant association was found between hPDI with PTH. In contrast, a significant positive association was found between overall PDI with PTH serum levels and an inverse association was found between uPDI and PTH serum levels. PTH indirectly induces bone resorption through osteoclasts as well as its receptors excited on osteoblasts (Bilezikian et al., 2008). Vitamin D is a well‐known beneficial nutrient for bone health. However, the beneficial effect of vitamin D on bone mass may depend on the level of intake and other factors. In our study, no significant association existed between 25‐hydroxy vitamin D concentrations and PDI, hPDI, and uPDI. In a cross‐sectional study, serum 25‐hydroxyvitamin D concentrations in vegans, but not in lacto‐ovo vegetarians, were slightly lower than those in omnivores (Xie et al., 2019). In another study, vegans had higher serum PTH concentration and lower 25(OH)‐D serum concentrations (Hansen et al., 2018).

Findings of the current study also suggested there is no significant association between PDI, hPDI, and uPDI and insulin resistance. A clinical trial study suggested that plant protein, as a part of a plant‐based diet, is associated with reductions in both body weight and insulin resistance (Kahleova et al., 2018). A study conducted by Esfandiari et al indicated there was no significant association between Mediterranean dietary pattern and healthy eating index with the development of insulin resistance. In contrast, individuals who adhered to the DASH dietary pattern have a lower risk of insulin resistance and its associated metabolic outcomes (Esfandiari et al., 2017). Decreased insulin resistance was also reported in prehypertensive individuals that adhered to 20‐ week DASH diet (Yazici et al., 2009). In another cross‐sectional study, vegetarian diet, in particular vegan diet, is inversely associated with insulin resistance, independent of body mass index (Cui et al., 2019). In contrast, no beneficial effect was observed on insulin sensitivity following 6 months DASH dietary pattern intervention, in both individuals with and without metabolic syndrome (Lien et al., 2007).

Our results showed that there is a significant effect of overall PDI on lowering insulin serum levels but there is an inverse association between uPDI and insulin serum levels. Also, no correlation was found between plant‐based dietary indicators and insulin sensitivity markers such as HOMA_IR and Quicki.

Prospective cohort studies suggested that dietary fiber specially insoluble fiber significantly reduces insulin resistance and developing type 2 diabetes (Weickert & Pfeiffer, 2018), and Olfert et al in their study indicated that vegetarian diets include vegan diets (no animal products), lacto‐ovi‐vegetarian diets (no animal meat, but milk and eggs), pesco‐vegetarian diets (fish), and semi‐vegetarian (Occasional consumption of meat) are associated with a reduced risk of diabetes (Agrawal et al., 2014).

Results of a narrative review suggested that the intake of polyphenols through plant‐based foods including whole grains, vegetables, fruits, coffee, tea, and nuts may be beneficial for both insulin resistance and T2D risk (Guasch‐Ferré et al., 2017). In contrast, some studies believe that there is no convincing evidence that soluble dietary fibers from fruit and vegetables might reduce the risk of diabetes (Weickert & Pfeiffer, 2018).

Several mechanisms justify our results. The plant‐based diet containing, in general, less calcium, less vitamin D, less iron, and less of other nutrients that have a positive influence on bone health (P. Burckhardt, 2016; McEvoy et al., 2012). However, it should be noted the main source of vitamin D is sunlight. This is maybe the reason that we were observed no association between PDI and 25‐hydroxy vitamin D. Plant foods rich in zinc, such as legumes, whole grains, seeds, and nuts, are also high in phytic acid, the main inhibitor of zinc bioavailability. On the other hand, bioavailability of zinc is enhanced by dietary protein, but plant sources of protein are also generally high in phytic acid (Harland & Oberleas, 1987). Furthermore, carotenoids may contribute to bone resorption via an antioxidant mechanism. In general, only a protein intake of less than 0.8 g/kg body weight is considered insufficient for bone health. It can be concluded that plant‐based diets often contain lower amounts of protein than those of other diets. Therefore, vegetarians might be risk candidates for osteoporosis (Peter Burckhardt et al., 2010). On the other hand, the plant‐based diet also has some characteristics that are positive for bone. The positive effect of fruit and vegetables can be partially explained by the calcium‐sparing effect of potassium (Lemann et al., 1989). In addition, this diet contains more potassium, vitamins, phytochemicals, antioxidants and bear a much lower acid load, both being reported as positive for bone (P. Burckhardt, 2016). All these negative and positive influences of the plant‐based diet may neutralize the effect of each other. Therefore, these reasons clarify our results that no associations were observed between PDI and bone turnover biomarkers.

Our research has many points of strength. To best our knowledge, this is the first time that the association between PDI and bone turnover is evaluated in older adults. In this paper, the food frequency questionnaire method was used to assess the dietary intake of patients that could reflect long‐term intake in adults. Moreover, we considered that the potential beneficial influences of a more plant‐based diet were independent of less healthy plant foods, for instance, sweets, sugary beverages, and refined grains, thereby the quality of plant‐based foods ingested is so important. Furthermore, we tried to adjust all possible potential confounders. However, this study does possess limitations, Because of cross‐sectional design, the possibility of residual confounding could not be ignored. The reliance on self‐reported methods to assess dietary intake was also a limitation due to measurement error and misclassification of participants. Another limitation was the small sample size that could affect the power of the study to identify a weak relationship.

## 5 CONCLUSION

In conclusion, data from this study in Iranian older adults demonstrated that more adherence to PDI and hPDI and less in uPDI may have a beneficial effect on biomarkers of bone, inflammation, and insulin thus preserving chronic diseases. To clarify, more research in large cohort studies and well‐designed clinical trials is needed.

## CONFLICT OF INTERESTS

The authors declare that they have no competing interests.

## AUTHORS CONTRIBUTIONS

Hossein Shahinfar: Conceptualization (Equal), Formal analysis (Equal), Software (Equal), Writing—original draft (Equal). Mohammad Reza Amini: Data curation (Equal). Nastaran Payande: Investigation (Equal). Sina naghshi: Writing—original draft (Equal). Fatemeh Sheikhhossein: Writing—original draft (Equal). Kurosh djafarian: Data curation (Supporting), Project administration (Equal), Validation (Lead), Writing—review and editing (Equal). Sakineh Shab‐Bidar: Conceptualization (Equal), Formal analysis (Equal), Writing—review and editing (Equal).

## ETHICAL APPROVAL

This study was conducted according to the guidelines laid down in the Declaration of Helsinki, and all procedures involving research study participants were approved by the ethics committee of Tehran University of Medical Sciences (Ethics Number: IR.TUMS.VCR.REC.1396.2307). Written informed consent was obtained from all subjects/patients.

## CONSENT FOR PUBLICATION

Participants were provided a study overview, and verbal consent was attained.

## Supporting information

Table S1

## Data Availability

The datasets generated or analyzed during the current study are not publicly available but are available from the corresponding author on reasonable request.
