# Supplementary material for: The link between plant‐based diet indices with biochemical markers of bone turn over, inflammation, and insulin in Iranian older adults
Source: Food Sci Nutr. 2021 Mar 29;9(6):3000–14. doi: 10.1002/fsn3.2258 (PMC8194905; doi:10.1002/fsn3.2258)
Supplement: Supplementary file 1 — Table S1 [file FSN3-9-3000-s001.docx]

| **Supplementary table 1. Examples of food items constituting the 18 food groups** | | | | |
| --- | --- | --- | --- | --- |
|  |  | **PDI** | **hPDI** | **uPDI** |
| **Plant Food Groups** |  |  |  |  |
| **Healthy** |  |  |  |  |
| **Whole grains** | Dark breads (e.g., barbari, sangak, taftun), bran breads, others | Positive scores | Positive scores | Reverse scores |
| **Fruits** | Melon, watermelon, honeydew melon, plums, prunes, apples, cherries, sour cherries, peaches, nectarine, pear, fig, date, grapes, kiwi, pomegranate, strawberry, banana, persimmon, berry, pineapple, oranges, dried fruits, others | Positive scores | Positive scores | Reverse scores |
| **Vegetables** | Cauliflower, carrot, tomato and its products, spinach, lettuce, cucumber, eggplant, onion, greens, green bean, green pea, squash, mushroom, pepper, corn, garlic, turnip, others | Positive scores | Positive scores | Reverse scores |
| **Nuts** | Almonds, peanut, walnut, pistachio, hazelnut, seeds, others | Positive scores | Positive scores | Reverse scores |
| **Legumes** | Lentils, split pea, beans, chick pea, fava bean, soy, others | Positive scores | Positive scores | Reverse scores |
| **Vegetable oils** | Oil-based salad dressing, vegetable oil used for cooking | Positive scores | Positive scores | Reverse scores |
| **Tea & Coffee** | Tea, coffee | Positive scores | Positive scores | Reverse scores |
| **Less healthy** |  |  |  |  |
| **Fruit juices** | Apple juice, orange juice, grapefruit juice, other fruit juice | Positive scores | Reverse scores | Positive scores |
| **Refined grains** | Lavash bread, baguette bread, rice, pasta, others | Positive scores | Reverse scores | Positive scores |
| **Potatoes** | French fries, baked or mashed potatoes, potato or corn chips | Positive scores | Reverse scores | Positive scores |
| **Sugar sweetened beverages** | Soft drinks, sugar sweetened beverages | Positive scores | Reverse scores | Positive scores |
| **Sweets and Desserts** | Cookies, cakes, biscuits, muffins, pies, chocolates, honey, jam, sugar cubes, sugar, candies, sweet tahini, others | Positive scores | Reverse scores | Positive scores |
| **Animal Food Groups** |  |  |  |  |
| **Animal fat** | Butter added to food, butter or lard used for cooking | Reverse scores | Reverse scores | Reverse scores |
| **Dairy** | Low-fat milk, skim milk, low-fat yogurt, cheese, Kashk, yogurt drink, High-fat milk, high-fat yogurt, cream cheese, cream, dairy fat, ice cream, others | Reverse scores | Reverse scores | Reverse scores |
| **Egg** | Eggs | Reverse scores | Reverse scores | Reverse scores |
| **Fish or Seafood** | Canned tuna, all other fishes, | Reverse scores | Reverse scores | Reverse scores |
| **Meat** | Beef and veal, lamb, minced meat, sausage, deli meat, hamburger, chicken, heart, kidney, liver, tongue, brain, offal, rennet | Reverse scores | Reverse scores | Reverse scores |
| **Misc. animal-based foods** | Pizza, chowder or cream soup, mayonnaise or other creamy salad dressing | Reverse scores | Reverse scores | Reverse scores |
